# Case Report: Pulmonary brucellosis presenting as multiple cavitary lung lesions on imaging

**DOI:** 10.3389/fmed.2026.1814731

**Published:** 2026-05-07

**Authors:** Tong Wang, Min Wang, Li Zhao, Guoke Tang, Lili Hou

**Affiliations:** 1 Department of Radiation Oncology, Lanzhou University, Lanzhou, Gansu, China; 2 Xincheng Hospital, Jiuquan Branch of Shanghai General Hospital, Jiuquan, Gansu, China; 3 Department of Orthopedics, Shanghai General Hospital, Shanghai Jiao Tong University, Shanghai, China; 4 Department of Respiratory and Critical Care Medicine, Shanghai General Hospital, Shanghai Jiao Tong University School of Medicine, Shanghai, China; 5 Department of Respiratory and Critical Care Medicine, Jiuquan Branch of Shanghai General Hospital, Jiuquan, Gansu, China

**Keywords:** case report, diagnosis, metagenomic next generation sequencing, pulmonary brucellosis, pulmonary cavity

## Abstract

Pulmonary brucellosis is a rare focal manifestation of human brucellosis with non-specific clinical features. Predominant imaging findings include pneumonia, pleural effusion, pulmonary nodules, abscesses, and interstitial changes. Multiple cavitary lesions are exceptionally rare. Herein, we report a case of bilateral multiple pulmonary cavities in a 76-year-old man with a 2-year history of intermittent cough, sputum production, and progressive dyspnea that acutely worsened 10 days prior to admission with intermittent fever, anorexia, and fatigue. Chest computed tomography (CT) revealed bilateral upper lobe irregular mass-like opacities and multiple nodules with heterogeneous density, punctate calcifications, and cavitation; multiple microcavitations in the right middle and lower lobes and the left lower lobe; and enlarged, calcified hilar and mediastinal lymph nodes. Metagenomic next-generation sequencing of bronchoalveolar lavage fluid identified *Brucella* species, which was confirmed by positive serology. After 3 days of doxycycline (0.1 g bid po) and rifampicin (0.6 g qd po), followed by 140 days of doxycycline (0.1 g bid po), rifapentine (0.6 g biw po), and levofloxacin (0.5 g qd po), along with silibinin meglumine tablets 0.1 g tid po for hepatoprotective therapy, the patient became afebrile with significant symptomatic improvement. Repeat chest CT demonstrated reduction in the right upper lobe consolidation/cavity and left upper lobe consolidation, resolution of the right lower lobe cavity, and complete resolution of the microcavitations. This case underscores that pulmonary brucellosis should be considered in the differential diagnosis of cavitary lung lesions in patients with livestock exposure and that prolonged combination antibiotic therapy can achieve favorable clinical and radiological outcomes.

## Introduction

Brucellosis, a zoonosis caused by *Brucella* spp., is primarily transmitted through direct inoculation, ingestion of unpasteurized dairy products, or inhalation of aerosolized organisms. Brucellosis is endemic in the Middle East, Asia, Africa, South and Central America, the Mediterranean, and the Caribbean (1–3). Over 170 countries and territories have reported cases of brucellosis. Outbreaks in Syria, Mexico, Peru, Argentina, and parts of Africa have been especially severe, causing substantial economic losses in the livestock industry and posing significant occupational risks to farmers, veterinarians, and abattoir worker (2, 3).

Brucellosis is a multisystem disease with highly variable clinical manifestations, most commonly involving the osteoarticular system (4, 5). Respiratory involvement is rare; when present, it typically causes cough, pleuritic pain, sputum production, dyspnea, or hemoptysis (6). On focused pulmonary examination, findings may include crackles, localized dullness to percussion, pleural rub, or dullness over the chest (6, 7). The predominant imaging features include pneumonia, pleural effusion, pulmonary nodules, mediastinal/thoracic lymph node enlargement, abscesses, and interstitial changes, which may occur individually or in combination (6). Multiple cavitary lung lesions remain exceedingly rare. Therefore, pulmonary involvement is frequently recognized late and may be misdiagnosed as tuberculosis (8, 9). Pulmonary brucellosis is inherently difficult to diagnose, and biotyping presents additional challenges. A definitive diagnosis requires isolation of *Brucella* spp. from the blood, pleural fluid, or sputum or a positive standard tube agglutination test (6). However, culture methods are slow and frequently yield false-negative results, delaying targeted therapy. Prior empirical use of broad-spectrum antibiotics may further reduce the yield.

Metagenomic next-generation sequencing (mNGS) has emerged as an effective diagnostic tool for bacterial diseases by offering high sensitivity, specificity, and accuracy in species identification across a wide range of clinical specimen, such as cerebrospinal fluid, plasma, respiratory secretions, bronchoalveolar lavage fluid (BALF), urine, stool, and fresh or formalin-fixed tissues. It has been widely used to detect infectious pathogens, particularly rare or emerging pathogens, demonstrating higher diagnostic efficacy than traditional methods (10–12). Here, we report a retrospective case of primary *Brucella* pneumonia in an elderly man, underscoring both the rarity of cavitary lung disease caused by *Brucella* spp. and the pivotal role of mNGS in establishing the diagnosis.

## Case description

A 76-year-old man presented with a 2-year history of intermittent cough, productive white frothy sputum, and progressively worsening breathlessness. The symptoms acutely exacerbated 10 days prior to admission, accompanied by fever, yellow purulent sputum, poor appetite, and fatigue. As oral ibuprofen provided no relief, the patient presented to our outpatient clinic. He denied having night sweats, chest pain, paroxysmal nocturnal dyspnea, or recent weight changes. He had experienced intermittent knee and lower back pain for 40 years that was relieved sporadically with ibuprofen or metamizole. The patient had hypertension for 6 years, treated with daily nifedipine tablets. He had a 42-pack-year smoking history. He is a farmer who has been in contact with cattle and sheep since childhood. He had no known allergies or family history of lung disease. He denied alcohol use, recent travel, or tuberculosis exposure.

The patient’s body temperature was 39.0 °C, blood pressure 154/86 mm Hg; heart rate 88 beats per minute; and oxygen saturation 93% on ambient air. The chest exhibited a barrel shape with widened intercostal spaces; breath sounds were coarse bilaterally, with a few moist crackles audible. All other physical examination results were normal.

Blood examination on 26 August 2025 revealed slightly elevated neutrophil count (6.32 × 10^3^/μL), C-reactive protein (CRP, 156.7 mg/L), and erythrocyte sedimentation rate (ESR, 101 mm/h). Hemoglobin was mildly reduced (114 g/L) (Table 1). Procalcitonin was slightly elevated (0.07 ng/mL). Tuberculin skin test, serum beta-D glucan, aspergillus galactomannan, gamma-interferon release assay, and PPD testing were negative. Renal and liver function tests and urinalysis results were normal, without occult blood. Nucleic acid testing of the sputum revealed infection with *Klebsiella pneumoniae* (*K. pneumonia*) and *Haemophilus influenzae*. Sputum remained negative for acid-fast bacilli and *Mycobacterium tuberculosis* nucleic acids on repeated testing. Serum tumor markers were negative.

**TABLE 1:** Changes in laboratory parameters before and after treatment.

Laboratory parameters	08-26-2025	08-31-2025	10-20-2025	01-21-2026
White blood cell (3.5–9.5 × 10^9^/L)	7.97	–	7.94	7.08
Neutrophils (1.8–6.3 × 10^9^/L)	6.32	–	4.67	4.87
Neutrophils percentage (%, 40%—5%)	79.3	–	58.9	68.7
Hemoglobin (130–175 g/L)	114	–	127	135
C-reactive protein (≤8 mg/L)	156.7	187.5	21.6	5.2
Procalcitonin (<0.05 ng/ml)	0.07	0.226	0.06	–
Erythrocyte sedimentation rate (0–15 mm/h)	101	101	54	10
Alanine aminotransferase (≤41 U/L)	32.5	–	11.4	10.3
Aspartate aminotransferase (≤37 U/L)	13.6	–	14.4	15.1

**FIGURE 1:**
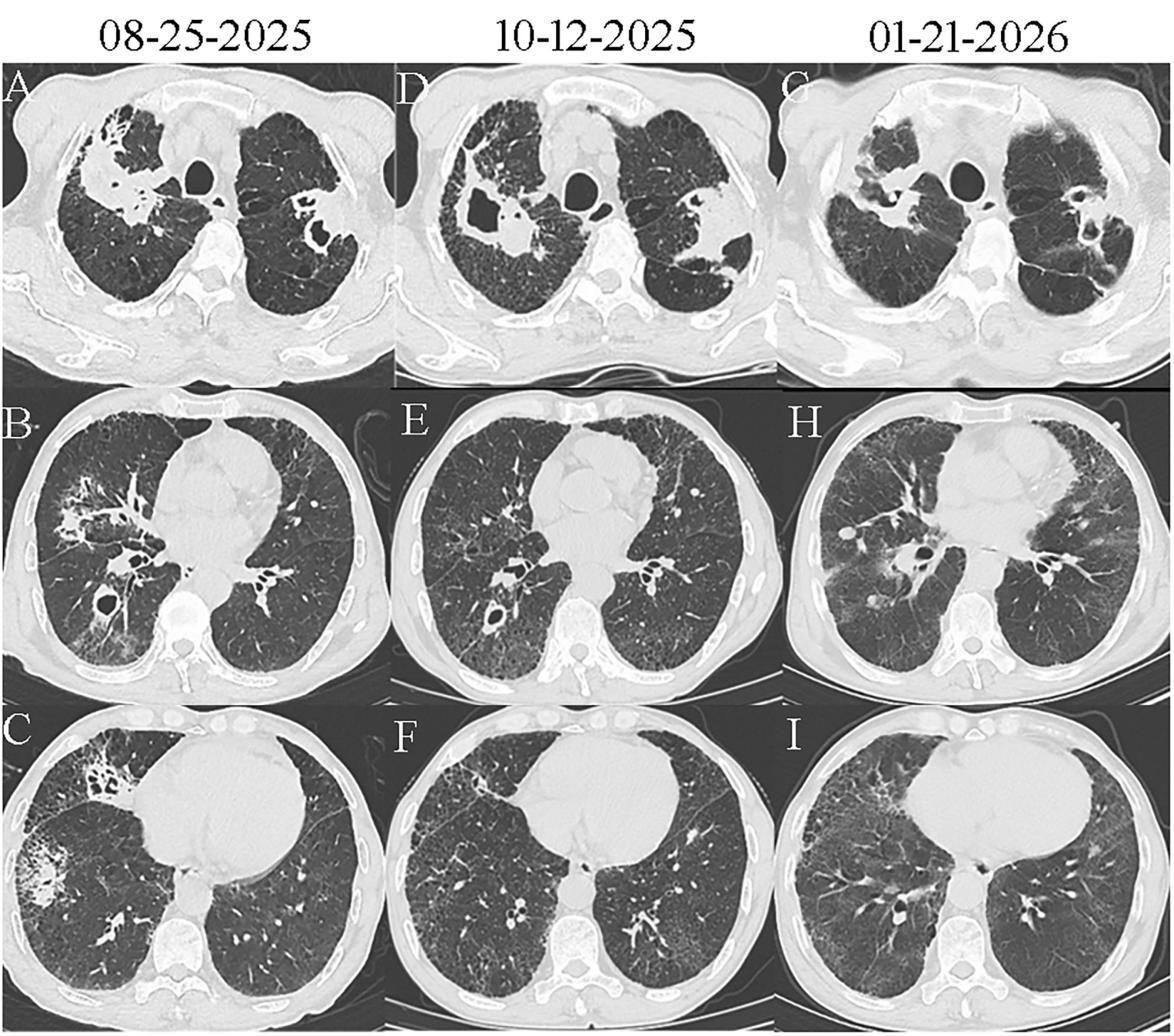
Pre-treatment chest computed tomography (CT) (the first column, 08-25-2025) revealed: **(A)** Consolidation of both upper lobes with cavity formation. **(B)** Consolidation in the right middle lobe and ground-glass opacities with cavitation in the right lower lobe. **(C)** Multi micro-cavities in the right middle and lower lobes. Post-treatment chest CT (the second column, 10-12-2025) showed: **(D)** The cavity in the right upper lobe had enlarged, whereas its consolidation had decreased; conversely, the left upper lobe cavity had diminished and its consolidation had increased. **(E)** Consolidation in the right middle lobe had cleared, the ground-glass opacities in the right lower lobe had resolved, and the associated cavity was smaller. **(F)** The multi micro-cavities previously seen in the right middle and lower lobes had almost completely resolved. Post-treatment chest CT (the third column, 01-21-2026): **(G)** Reduction in the size of both the right upper lobe consolidation/cavity and the left upper lobe consolidation. **(H)** The associated cavity in the right lower lobe had resolved. **(I)** The multi micro-cavities previously seen in the right middle and lower lobes had completely resolved.

Chest computed tomography (CT) on 25 August 2025 showed that both upper lobes exhibited irregular mass-like opacities and multiple nodules, some with heterogeneous density, punctate calcifications, and cavitation. The right middle and lower lobes demonstrated cavities and multiple micro-cavitations. Enlarged, calcified lymph nodes were observed in both the hila and mediastinum, suggesting changes in secondary pulmonary tuberculosis (Figure 1A–C).

**FIGURE 2:**
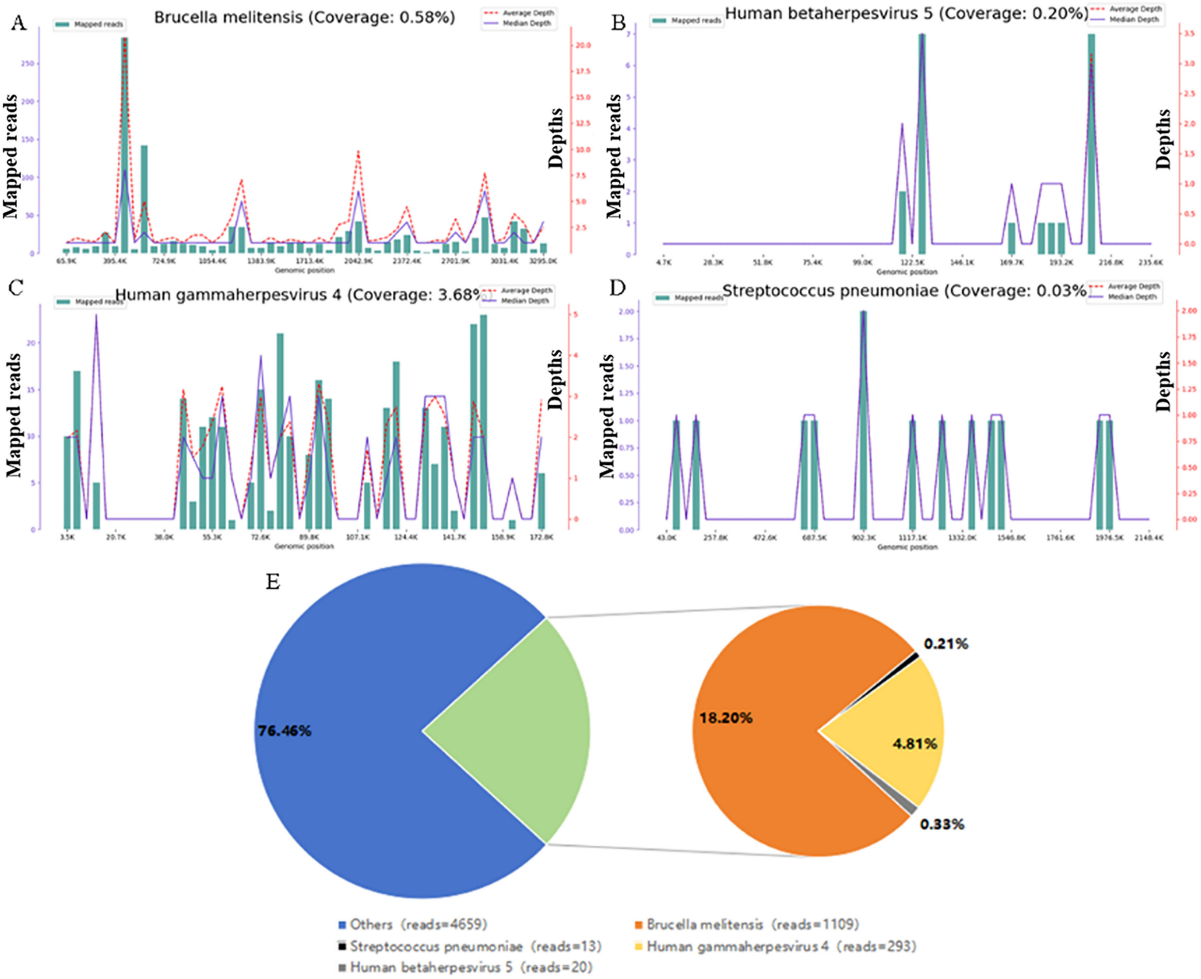
The patient’s bronchoalveolar lavage fluid (BALF) metagenomic next-generation sequencing (mNGS) result. Genome coverage graphs of pathogens: *Brucella melitensis* **(A)**, Human betaherpesvirus 5 **(B)**, Human gammaherpesvirus 4 **(C)**, *Streptococcus pneumoniae* **(D**). The abscissa denotes the genome position. The left ordinate shows the number of pathogen reads aligned to the genome location (mapped reads, green bars), and the right ordinate indicates the depth at the corresponding position (the average sequencing depth, red dashed line; median depths, purple line). **(E)** The proportion of pathogen sequences among the microbes detected in the patient’s BALF sample. The orange section is *Brucella melitensis* (18.20%), the gold section is Human gammaherpesvirus 4 (4.81%), the gray section is Human betaherpesvirus 5 (0.33%), the black section is *Streptococcus pneumoniae* (0.21%), and the blue section is other microorganisms.

**FIGURE 3:**
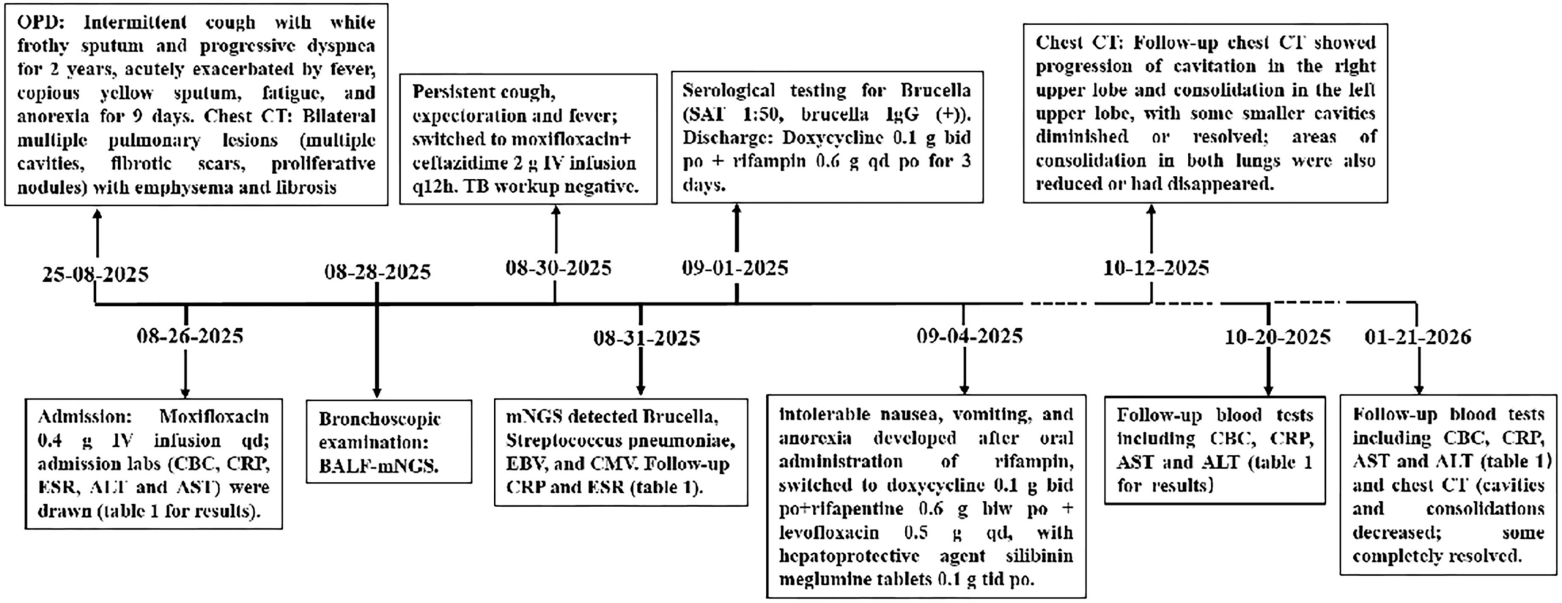
Timeline of the clinical course including symptom onset, initial presentation, empirical antibiotic therapy, mNGS diagnosis, targeted therapy initiation and follow-up assessments. OPIJ, outpatient department; CHC, complete blood Count; CRP. C-reactive protein; CT, computed tomography; ESR, erythrocyte sedimentation rate; BALF, bronchoalveolar lavage fluid; m-NGS, metagenomic next-generation sequencing; EBV, Epstein-Barr virus; CMV, cytomegalovirus; qd, quaque die (once daily); biw, bis in week (twice weekly); bid, bis in die (twice a day): tid, ter in die (three times a day); PO, per os: IV, intravenous; SAT, serum agglutination test: IgG, immunoglobulin G; ALT, alanine aminotransferase: AST, aspartate aminotransferase

The patient received moxifloxacin (0.4 g intravenously once daily) for 4 days, without significant clinical improvement. Laboratory reassessment on 31 August 2025 demonstrated worsening inflammation: CRP increased to 187.5 mg/L and procalcitonin to 0.226 ng/mL, whereas ESR remained unchanged at 101 mm/h. Therefore, ceftazidime (2 g every 12 h intravenously) was added to the antibiotic regimen. Following 3 days of combination therapy, his maximum body temperature decreased to 38 °C, yet his symptoms showed no significant improvement. mNGS of BALF on 31 August 2025 detected 18207 reads mapping to *Brucella*, including 216 assigned to *Brucella melitensis* (24.27% relative abundance); other pathogens included *Streptococcus pneumoniae* (192 reads, 1.01%), Epstein-Barr virus (5 reads, 0.56%), and cytomegalovirus (1 read, 0.11%) (Figure 2). Serological tests on 1 September 2025 for brucellosis showed positive results: Rose Bengal plate agglutination was positive, serum agglutination test (SAT) titer was 1:50, and Brucella IgG antibody was positive. Moxifloxacin and ceftazidime were discontinued, and oral doxycycline (0.1 g twice daily) combined with rifampicin (0.6 g once daily) was initiated. However, after 3 days, the patient developed intolerable nausea, vomiting, and other adverse effects. The regimen was subsequently adjusted to doxycycline (0.1 g twice daily), rifapentine (0.6 g twice weekly), and levofloxacin (0.5 g once daily), along with silibinin meglumine tablets (0.1 g three times daily) for hepatoprotective therapy, all administered orally. The patient tolerated the new regimen without any adverse reactions. After 38 days on this regimen, the patient became afebrile and reported marked improvements in cough, sputum production, and fatigue. Laboratory data, including neutrophil count, CRP, and ESR on 20 October 2025 were markedly lower than those in the previous tests (Table 1). Repeat chest CT on 10 December 2025 showed progression of cavitation in the right upper lobe and consolidation in the left upper lobe, while some smaller cavities had diminished or resolved; areas of consolidation in both lungs were also reduced or had disappeared (Figure 1D–F). After completing 140 days of doxycycline, rifapentine, and levofloxacin, the patient’s symptoms showed further significant improvement, with CRP and ESR returning to normal levels on 21 January 2026 (Table 1). After re-examination, chest CT on 21 January 2026 showed a reduction in the size of both the right upper lobe consolidation/cavity and the left upper lobe consolidation, resolution of the associated cavity in the right lower lobe, and complete resolution of the multiple microcavities previously observed in the right middle and lower lobes (Figure 1G–I). The clinical course is summarized in a timeline (Figure 3).

## Discussion

Brucellosis is a zoonotic infection caused by gram-negative bacteria of the genus *Brucella*. The species most frequently implicated in human disease are, in descending order of clinical importance, *B. melitensis*, *B. abortus* and *B. suis*, whereas *B. canis* is encountered less often. Transmission occurs via direct contact with infected animals, ingestion of unpasteurized dairy products, or inhalation of aerolized organisms. Erdem et al. identified raw dairy consumption as the dominant risk factor (61.9%), with hematogenous spread as the main route to the lungs (7).

Pulmonary involvement is uncommon in brucellosis, and cavitary pulmonary brucellosis is extremely rare. In radiology, a cavity signifies a gas-filled, wall-bordered lucency within a consolidation, mass, or nodule, pathologically reflecting evacuation of necrotic debris via the bronchial tree and radiologically appearing as a variable-thickness-walled focus that may contain an air-fluid level. In common bacterial infections, whether caused by gram-positive or gram-negative species, pulmonary cavities develop via two routes: organisms may ascend the bronchial tree, evade host defenses, and generate necrotizing pneumonia or abscesses, or they may be delivered to the lungs as septic pulmonary emboli during bloodstream infections.

Liquefactive necrosis is the key pathological basis for forming thick-wall cavities with air-fluid levels (8). Pyogenic pathogens including *K. pneumoniae*, *Staphylococcus aureus*, the *Streptococcus anginosus* group, and *H. influenzae* typically possess this characteristic. However, the clinical, microbiological, and radiological features of the present case did not support these common pathogens. The patient lacked typical risk factors for *K. pneumoniae* infection such as diabetes mellitus, alcoholism, malignancy, or liver abscess. Although sputum DNA testing was positive for *K. pneumoniae* and *H. influenzae*, BALF specimens were negative by mNGS and culture, suggesting oropharyngeal colonizer contamination of these bacteria. Furthermore, *K. pneumoniae* pneumonia typically presents with bulging fissure sign or lobar consolidation on chest CT, which was not observed in the present case.

Additionally, *S. pneumoniae* was identified in BALF mNGS but was not regarded as the primary causative pathogen. *Pneumococcal pneumonia* typically presents on chest CT as lobar consolidation with air bronchograms (13). In necrotizing pneumonia, multiple small, irregular lucencies or thin-walled cavities appear within the consolidated area, which progressively coalesce to form larger air-fluid cavities (13, 14). These characteristic findings differ markedly from the lung mass and pulmonary nodules with internal calcification and without air-fluid levels observed in the present case.

*Mycobacterium tuberculosis*, *Mycobacterium bovis*, and *Aspergillus* species induce coagulative necrosis, typically resulting in dry, non-fluid-filled cavities. In the present case, a negative galactomannan antigen test and the absence of immunosuppression rendered invasive pulmonary aspergillosis unlikely. Notably, although *Brucella* primarily causes granulomatous inflammation, it can also induce pulmonary cavity formation through the release of inflammatory mediators, leading to lung tissue fibrosis and scarring (15). This mechanism resembles that of tuberculosis, making cavitation in brucellar pneumonia pathologically plausible but rarely reported clinically.

Chest CT in the present case revealed bilateral pulmonary consolidation with multiple thick-walled cavities and multiple microcavities. To the best of our knowledge, this is one of the very few reported cases of multiple bilateral thick-walled cavities accompanied by multiple microcavities. This radiological pattern may reflect the distinctive pathological process of chronic granulomatous inflammation with organizing pneumonia characteristic of brucellar infection, suggesting that *Brucella* should be included in the differential diagnosis when patients present with “tuberculosis-like” cavitary lesions; however, microbiological evidence does not support tuberculosis, particularly in those with a history of livestock contact or travel to endemic areas.

A definitive diagnosis of pulmonary brucellosis requires the integration of epidemiological exposure, compatible clinical findings, and confirmatory laboratory evidence. Due to its non-specific presentation, brucellar pneumonia is frequently diagnosed late and may be misdiagnosed as tuberculosis or non-tuberculous mycobacterial lung disease. The median interval between symptom onset and first medical consultation has been reported as 4 weeks (range 0.3–30) (6). The patient in the present case had a delayed presentation of at least 4 weeks. Chest CT revealed long-standing disease, but he did not previously seek medical attention.

Isolation of *Brucella* spp. from sputum, blood, bone marrow, cerebrospinal fluid, and pleural or ascitic fluid remains the gold standard for diagnosis. A diagnosis of brucellosis is established by a serum agglutination test (SAT) titer of 1:100++or greater, or a titer of 1:50++ or greater in patients with persistent clinical symptoms for more than 1 year (16). Blood cultures have a positivity rate of 28%, whereas tissue cultures are positive in approximately 20.2% of cases (11). Empirical broad-spectrum antibiotic therapy can further reduce the blood culture yield (11). mNGS can theoretically detect all DNA-containing microorganisms in a sample, including *Brucella* (11, 17). This culture-independent, unbiased approach offers high sensitivity, is minimally affected by prior antibiotic use, and enables species-level identification (18). However, pulmonary brucellosis diagnosed by *Brucella* detection in BALF using mNGS has only been reported sporadically, and the reasons remain unclear. In the present case, a large number of *Brucella* reads were detected using mNGS, and partial sequencing identified *B. melitensis*.

Pulmonary brucellosis is treated with the same antibiotics and combination regimens as acute uncomplicated brucellosis. Commonly used first-line antibiotics include tetracyclines (particularly doxycycline), rifamycins, and aminoglycosides. For patients who cannot use first-line agents due to drug allergy, availability issues, or other reasons, or for whom these agents are ineffective, second-line drugs such as rifapentine and levofloxacin may be used. Early initiation of antibiotics is associated with better outcomes; if effective therapy is started within the first week of infection, cure rates exceed 90%. Our patient initially received levofloxacin and ceftazidime with no improvement in clinical symptoms or inflammatory markers (CRP and ESR), indicating that *K. pneumoniae* and *H. influenzae* detected by sputum nucleic acid testing represented normal flora rather than true pathogens.

The patient experienced significant adverse effects, such as nausea and vomiting, with the first-line regimen of doxycycline and rifampicin. Consequently, a second-line regimen was administered orally, consisting of doxycycline, rifapentine, and levofloxacin. Notably, after 38 days of treatment with this second-line regimen, a follow-up chest CT scan revealed progression of cavitation in the right upper lobe and consolidation in the left upper lobe. This was possibly due to immune reconstitution inflammatory syndrome (19) or a Jarisch-Herxheimer reaction (20), as the patient’s symptoms, physical signs, and laboratory parameters improved, which supports the diagnosis of pulmonary brucellosis and the efficacy of the treatment. Possibly due to the concurrent use of hepatoprotective agents, no significant liver function impairment occurred during the course of medication.

This report has several limitations. First, as a single case report, the generalizability of the findings is limited; nevertheless, documenting and compiling such cases remains valuable. Second, the recalcitrant cell wall of *Brucella* impedes efficient DNA extraction, likely explaining the paucity of *B. melitensis* sequences detected in the present case. Furthermore, mNGS-based identification provides no antimicrobial susceptibility information, thereby complicating resistance monitoring and restricting clinicians to empirical therapy. Third, owing to patient reluctance to undergo repeat bronchoscopy and economic constraints, treatment response was evaluated clinically and radiologically without repeat BALF mNGS. Although clinical and radiological resolution was achieved, the absence of microbiological confirmation precludes definitive assessment of pathogen eradication. Fourth, chronic obstructive pulmonary disease (COPD) is a likely comorbidity given the patient’s smoking history, chronic respiratory symptoms, and barrel chest. However, spirometry could not be performed during the active infectious phase and due to economic constraints. Comprehensive COPD evaluation, including pulmonary function testing, is recommended once the infection is controlled. Despite these limitations, this case offers valuable clinical insights into distinguishing cavitary pulmonary infections and optimizing treatment strategies for brucellosis pneumonia.

## Conclusion

Pulmonary brucellosis can produce cavitary pulmonary lesions on imaging and should be included in the differential diagnosis of respiratory infections, especially in endemic areas or when there is epidemiological evidence of exposure to *Brucella* spp. Early recognition, use of mNGS when conventional methods are inconclusive, and prolonged combination antibiotic therapy can lead to favorable clinical and radiological outcomes.

## Patient perspective

The patient is a 76-year-old farmer who has worked with cattle and sheep his whole life. When he developed a persistent cough and fever that did not improve with initial antibiotics, he became very worried. He was relieved when the doctors finally diagnosed him with brucellosis. Although the first treatment caused nausea and vomiting, the doctors adjusted his medications, and he tolerated the new regimen well. Over time, his symptoms gradually improved. He is truly grateful to the medical team for their care and support throughout his treatment.

## Data Availability

The data supporting the conclusions of this article will be made available by the authors on reasonable request.

## References

[1] Dadar M Alamian S Brangsch H Elbadawy M Elkharsawi A Neubauer H et al.. Determination of virulence-associated genes and antimicrobial resistance profiles in Brucella isolates recovered from humans and animals in Iran using NGS technology. *Pathogens.* (2023) 12:82. 10.3390/pathogens12010082 36678430 PMC9865427

[2] Pappas G Papadimitriou P Akritidis N Christou L Tsianos E. The new global map of human brucellosis. *Lancet Infect Dis.* (2006) 6:91–9. 10.1016/S1473-3099(06)70382-6 16439329

[3] Cross A Baldwin V Roy S Essex-Lopresti A Prior J Harmer N. Zoonoses under our noses. *Microbes Infect.* (2019) 21:10–9. 10.1016/j.micinf.2018.06.001 29913297 PMC6386771

[4] Wu X Wang R. Multimodal magnetic resonance imaging and radiomics in Brucella spondylitis: diagnostic applications and differential diagnosis. *J Multidiscip Healthc.* (2025) 18:6091–101. 10.2147/JMDH.S536880 41030906 PMC12477277

[5] Ali Adam A Sheikh Hassan M Adam Osman A. Spinal brucellosis causing spondylodiscitis. *Ann Med Surg.* (2022) 82:104782. 10.1016/j.amsu.2022.104782 36268353 PMC9577941

[6] Solera J Solís García Del Pozo J. Treatment of pulmonary brucellosis: a systematic review. *Expert Rev Anti Infect Ther.* (2017) 15:33–42. 10.1080/14787210.2017.1254042 27790937

[7] Erdem H Inan A Elaldi N Tekin R Gulsun S Ataman-Hatipoglu C et al.. Respiratory system involvement in brucellosis: the results of the Kardelen study. *Chest.* (2014) 145:87–94. 10.1378/chest.13-0240 23907372

[8] Gadkowski L Stout J. Cavitary pulmonary disease. *Clin Microbiol Rev.* (2008) 21:305–33. 10.1128/CMR.00060-07 18400799 PMC2292573

[9] Chapra A Kazman R Habib M Ashour A Sadek M Shaukat A. Brucella pneumonia mimicking pulmonary tuberculosis: a case report. *Clin Case Rep.* (2025) 13:e70387. 10.1002/ccr3.70387 40226234 PMC11985891

[10] Zhu Y Sun M Chen B Liu X Yang G Li X. Diagnostic value of cerebrospinal fluid metagenomics next-generation sequencing in neurobrucellosis in children. *Pediatr Infect Dis J.* (2025) 44:e329–32. 10.1097/INF.0000000000004845 40440710 PMC12333518

[11] Yao X Zhao G Wang L Jia C. Study on the value of second-generation sequencing technology in the clinical diagnosis of osteoarticular brucellosis. *J Orthop Res.* (2024) 42:2327–35. 10.1002/jor.25867 38722074

[12] Miller S Chiu C. The role of metagenomics and next-generation sequencing in infectious disease diagnosis. *Clin Chem.* (2021) 68:115–24. 10.1093/clinchem/hvab173 34969106

[13] de La Porte des Vaux C Sainte-Rose V Le Turnier P Djossou F Nacher M Zappa M et al.. Chest CT findings in community-acquired pneumonia due to *Coxiella burnetii* (Q fever) compared to Streptococcus pneumoniae, a cross sectional study in French Guiana, 2013-2017. *Travel Med Infect Dis.* (2024) 57:102679. 10.1016/j.tmaid.2023.102679 38135242

[14] Masters I Isles A Grimwood K. Necrotizing pneumonia: an emerging problem in children? *Pneumonia.* (2017) 9:11. 10.1186/s41479-017-0035-0 28770121 PMC5525269

[15] Fu Y Wang Z Lu B Zhao S Zhang Y Zhao Z et al.. Immune response and differentially expressed proteins in the lung tissue of BALB/c mice challenged by aerosolized Brucella melitensis 5. *J Int Med Res.* (2018) 46:4740–52. 10.1177/0300060518799879 30282518 PMC6259401

[16] General Office of the National Health Commission, Comprehensive Department of the National Administration of Traditional Chinese Medicine. Diagnosis and treatment scheme for Brucellosis (2023 Edition). *Chinese J Infect Control.* (2024) 23:661–4. 10.12138/j.issn.1671-9638.20245429

[17] Zhao Y Zhang W Zhang X. Application of metagenomic next-generation sequencing in the diagnosis of infectious diseases. *Front Cell Infect Microbiol.* (2024) 14:1458316. 10.3389/fcimb.2024.1458316 39619659 PMC11604630

[18] Mu X Luo H Li H Chen S Han Y Zhang L et al.. Pathogen detection and antibiotic use in granulomatous lobular mastitis: a comparison of mNGS and culture. *Front Cell Infect Microbiol.* (2025) 15:1570776. 10.3389/fcimb.2025.1570776 40568706 PMC12188451

[19] Qu C Xu N Niu D Wen S Yang H Wang S et al.. Case report: suspected case of brucella-associated immune reconstitution inflammatory syndrome. *Front Immunol.* (2022) 13:923341. 10.3389/fimmu.2022.923341 35935931 PMC9353035

[20] Habeeb Y Al-Najdi A Sadek S Al-Onaizi E. Paediatric neurobrucellosis: case report and literature review. *J Infect.* (1998) 37:59–62. 10.1016/s0163-4453(98)90647-8 9733381

